# Context matters in implementation science: a scoping review of determinant frameworks that describe contextual determinants for implementation outcomes

**DOI:** 10.1186/s12913-019-4015-3

**Published:** 2019-03-25

**Authors:** Per Nilsen, Susanne Bernhardsson

**Affiliations:** 1 Department of Medical and Health Sciences, Division of Community Medicine, Linköping University, SE-581 83 Linköping, Sweden; 2 Närhälsan Research and Development Primary Health Care, Region Västra Götaland, Gothenburg, Sweden; 3 Institute of Neuroscience and Physiology, Department of Health and Rehabilitation, Unit of Physiotherapy, University of Gothenburg, The Sahlgrenska Academy, Gothenburg, Sweden

**Keywords:** Context, Determinants, Barriers, Frameworks, Implementation

## Abstract

**Background:**

The relevance of context in implementation science is reflected in the numerous theories, frameworks, models and taxonomies that have been proposed to analyse determinants of implementation (in this paper referred to as determinant frameworks). This scoping review aimed to investigate and map how determinant frameworks used in implementation science were developed, what terms are used for contextual determinants for implementation, how the context is conceptualized, and which context dimensions that can be discerned.

**Methods:**

A scoping review was conducted. MEDLINE and EMBASE were searched from inception to October 2017, and supplemented with implementation science text books and known published overviews. Publications in English that described a determinant framework (theory, model, taxonomy or checklist), of which context was one determinant, were eligible. Screening and inclusion were done in duplicate. Extracted data were analysed to address the study aims. A qualitative content analysis with an inductive approach was carried out concerning the development and core context dimensions of the frameworks. The review is reported according to the PRISMA guidelines.

**Results:**

The database searches yielded a total of 1113 publications, of which 67 were considered potentially relevant based on the predetermined eligibility criteria, and retrieved in full text. Seventeen unique determinant frameworks were identified and included. Most were developed based on the literature and/or the developers’ implementation experiences. Six of the frameworks explicitly referred to “context”, but only four frameworks provided a specific definition of the concept. Instead, context was defined indirectly by description of various categories and sub-categories that together made up the context. Twelve context dimensions were identified, pertaining to different aggregation levels. The most widely addressed context dimensions were organizational support, financial resources, social relations and support, and leadership.

**Conclusions:**

The findings suggest variation with regard to how the frameworks were developed and considerable inconsistency in terms used for contextual determinants, how context is conceptualized, and which contextual determinants are accounted for in frameworks used in implementation science. Common context dimensions were identified, which can facilitate research that incorporates a theory of context, i.e. assumptions about how different dimensions may influence each other and affect implementation outcomes. A thoughtful application of the concept and a more consistent terminology would enhance transparency, simplify communication among researchers, and facilitate comparison across studies.

**Electronic supplementary material:**

The online version of this article (10.1186/s12913-019-4015-3) contains supplementary material, which is available to authorized users.

## Background

The term “context” is derived from the Latin *cum* (“with” or “together”) and *texere* (“to weave”). Understanding what happens when an evidence-based practice, e.g. an intervention, programme, method or service, is “woven together” with a team, department or organization is important to better address implementation challenges in health care and other settings. Accounting for the influence of context is necessary to explain how or why certain implementation outcomes are achieved, and failure to do so may limit the generalizability of study findings to different settings or circumstances. Context is considered responsible for study-to-study variations in outcomes [1–9].

The relevance of the context in implementation science is reflected in the numerous theories, frameworks, models and taxonomies (referred in this paper to as frameworks) that are applied to analyse barriers and facilitators concerning various implementation outcomes [10]. Frameworks such as Promoting Action on Research Implementation in Health Services (PARIHS) [11, 12] and Theoretical Domains Framework (TDF) [13] explicitly refer to context as one of several determinants; other frameworks do not explicitly mention context. Instead, many other terms referring to the same or similar concept are in use, e.g. “environmental factors” [14] and “inner setting” and “outer setting” [15]. Terms such as “context”, “setting” and “environment” are often used interchangeably in implementation science and other research fields [8].

Regardless of which terms are used, it is not known whether these determinant frameworks conceptualize context in a similar way and describe the same context dimensions or to what extent they encompass different dimensions of the context. Lack of conceptual and terminological clarity and consistency makes it difficult for implementation researchers to identify the most relevant context dimensions for any given study. If neglected dimensions are causally significant for implementation outcomes, their omission may create problems in interpreting and applying the findings.

Some of these determinant frameworks are widely used in implementation science [16], which means that context as understood in these frameworks may have considerable impact on how the concept is studied de facto. No previous study has investigated determinant frameworks in terms of how they define or describe context and what might be a core set of contextual determinants that most frameworks account for. Therefore, the aim of this scoping review was to identify and examine determinant frameworks used in implementation science to address four issues: how were the frameworks developed, what terms do they use to denote contextual determinants for implementation, how is the context conceptualized, and which context dimensions are applied across the frameworks. Greater conceptual and terminological clarity and consistency may enhance transparency, improve communication among researchers, and facilitate exchange of data and comparative evaluations.

## Methods

### Approach

To address the study aims, a scoping review was undertaken to identify determinant frameworks that describe determinants, including those related to context, that may influence implementation outcomes, i.e. contextual determinants. Determinant frameworks are frameworks which have a descriptive purpose by pointing to factors believed or found to influence implementation outcomes. They do not specify the mechanisms of change; they are typically more like checklists of factors that influence implementation outcomes. They can be referred to as models, theories, checklists and taxonomies because the terminology is inconsistent [10].

A scoping review methodology was chosen because it allows for synthesis of findings across a range of study types and designs and provides a broad overview of a topic [17, 18]. Unlike systematic reviews, which address precise questions (e.g. the effectiveness of a particular type of intervention), scoping reviews can be used to map and clarify key concepts underpinning a research area [19]. To ensure that no published determinant framework would be missed, database searches were complemented with examination of textbooks in implementation science and studies that have presented comprehensive overviews of implementation theories, frameworks, models, checklists or taxonomies. The research questions and inclusion and exclusion criteria were established before the review was conducted. Although not always applicable, conduct and reporting of the review was guided by the Preferred Reporting Items for Systematic Reviews and Meta-Analyses (PRISMA) guidelines [20]. Because all data were publicly available, ethical review board approval was not necessary. The review protocol was not registered.

### Eligibility criteria

To be included in the review, studies in the English-speaking literature were required to report a determinant framework that described different determinants for implementation outcomes, including contextual determinants, in implementation of health care practices, from primary to tertiary care. Peer-reviewed scientific articles, as well as text books, were eligible for inclusion. A generic definition of implementation context was applied in this review. Contextual determinants were considered those determinants in the determinant frameworks that were not attributed to or associated with: the practice being implemented (e.g. an evidence-based practice); individual characteristics of the adopters (e.g. health care practitioners’ attitudes, beliefs and motivation concerning this practice); or the strategies used to support the implementation.

Determinants were defined as factors believed or empirically shown to influence implementation outcomes. Many terms are used for determinants, including barriers, hinders, obstacles, impediments, enablers, and facilitators. Implementation outcomes were defined broadly in terms of behaviours and adherence, adoption, uptake, or use concerning practices of relevance for achieving a more evidence-based health care practice [21].

We excluded theories which describe causal mechanisms of how various determinants may influence implementation outcomes. We also excluded theoretical approaches developed and used in fields other than implementation science, e.g. psychology, sociology, organizational theory and political science. Further, we excluded so-called process models, which describe the research-to-practice path and/or guide the implementation process rather than describe determinants of implementation outcomes [10].

Determinant frameworks with limited generalizability were excluded; for instance, those that focused on a specific health issue (e.g. diabetes), a specific population or patient group (e.g. HIV-positive persons), a specific intervention (e.g. brief alcohol interventions), and/or were generated to describe or structure the results of a single empirical study. We also excluded studies that only described applications of frameworks, because our aim was to identify studies that focused on describing and detailing the contents of the determinant frameworks (including the contextual determinants).

We also excluded community, public health and school settings, governance, health care priority settings and resource allocation, public policy, occupational health, workplace settings, and implementation of models of care. No study design limitations were applied, with the exception of study protocols.

### Search strategy

Preliminary searches were done in MEDLINE to identify search terms. MEDLINE and EMBASE were searched from inception to October 2017. These two databases were considered the most relevant for this review, and likely to cover the vast majority of determinant frameworks intended for use in health care settings. A comprehensive search strategy was developed for MEDLINE with support from a medical librarian, and subsequently adapted to the other database (Additional file 1). The search strategy combined search terms with medical subject headings and focused on identifying publications on determinant frameworks.

To supplement the database search, three additional sources were used. Reference lists in publications included for full-text review were screened to identify eligible frameworks. Nine textbooks that provided comprehensive overviews of research regarding implementation science were reviewed [22–30]. These textbooks were reviewed because they are written by influential implementation scientists and the authors of this review have them in their possession. Lastly, five comprehensive overviews of theoretical approaches in implementation science were examined [16, 31–34]. The authors teach implementation science theory at several Swedish universities, and are familiar with these sources as part of their teaching.

### Study selection

Both authors independently screened titles and abstracts and selected studies for potential inclusion in the study, applying the predefined inclusion and exclusion criteria. Both authors then read the full texts of these articles to assess eligibility for final inclusion. Any disagreement between the authors regarding eligibility was resolved in consensus.

### Data extraction

Data were collected on the following study characteristics: (1) authors; (2) publication year; (3) what was the desired outcome, as described in the framework?; (4) how were the determinants identified in the framework? (i.e. how was the framework developed?); (5) which determinant categories were described in the framework; (6) which of the determinant categories were associated with contextual determinants and/or are labelled “context”?; and (7) which contextual determinant categories and sub-categories did the framework include? Data extraction was done primarily by one reviewer (PN) and verified by the other (SB).

### Data analysis

Extracted data were analysed to address the four study aims. A qualitative content analysis with an inductive approach was carried out concerning how the frameworks were developed [35]. Qualitative content analysis is a method of analysing written, verbal or visual communication messages [36], with the aim of attaining a condensed and broad description of the phenomenon under study. The analytical process includes coding, creating categories and abstraction. The inductive approach means that the analysis is driven by the data and no a priori codes or categories are used. Terms that were used in the frameworks to denote contextual determinants for implementation were coded with regard to whether the framework referred explicitly to “context” or whether it used other terms to denote contextual determinants. Contextual determinants described in a framework were categorized into different context dimensions. We use the term context dimension(s) for our categorization of the contextual determinants (categories and sub-categories) described in the determinant frameworks. Conceptualization of context was analysed in relation to whether the framework provided explicit definitions of context or whether the concept was defined or understood by means of describing a number of contextual determinants.

Summary statistics (i.e. frequencies) were used to describe the number of frameworks that were developed in different ways, the number of frameworks that referred to “context”, the number of frameworks that provided explicit definitions of context, and the number of frameworks that addressed the various context dimensions that emerged from the analysis.

## Results

### Identification of determinant frameworks

Twenty-two relevant publications were identified, describing 17 unique determinant frameworks (Table 1). Database searches yielded a total of 1113 publications, of which 67 were considered potentially relevant and retrieved in full text. The searches yielded three publications each describing a unique determinant framework: Cabana et al. [37]; Cane et al. [13] (TDF); and Harvey and Kitson [38] (PARIHS). Seven publications were excluded because they did not describe a determinant framework, and one publication was excluded because the setting was not health care.

**Table 1:** Included determinant frameworks

Source	What is implemented and/or what is the desired outcome?	Development of the framework: how were the determinants identified in the framework?	Determinant categories (underlined categories are associated with contextual determinants and/or are labelled “context”)	Contextual determinants: categories and examples of sub-categories in the framework
PARIHS: Kitson et al., 1998 [11], Rycroft-Malone, 2010 [12]; i-PARIHS: Harvey and Kitson, 2016 [38]	Effective practice	PARIHS was “developed inductively from the originators’ experience as change agents and researchers” ([12], p. 111), followed by conceptual work and empirical studies. I-PARIHS was developed based on research applying PARIHS and to account for critiques and evaluations of the framework by other researchers	PARIHS, 3 categories (1 relates to contextual influences): • Evidence • Facilitation • Context i-PARIHS, 4 categories (2 relate to contextual influences): • Innovation • Facilitation • Recipients • Context Categories are referred to as “elements” and sub-categories as “sub-elements”	PARIHS: • Context: culture (including values concerning innovation, power and authority, allocation of human, financial and equipment resources, rewards/recognition); leadership (including type of leadership, role clarity, teamwork, organizational structures, decision-making processes, approach to learning); evaluation (including feedback on individual, team and system performance) The category Evidence in PARIHS concerns characteristics of the evidence (including research and clinical experience), but also includes patient influences i-PARIHS: • Context (local level): culture; formal and informal leadership; evaluation of innovation and change; learning environment. Context (organizational level): senior leadership and management support; culture; structure and systems; absorptive capacity. Context (external health system level): policy drivers and priorities; regulatory frameworks; environmental (in) stability; inter-organizational networks and relationships • Recipients: collaboration and teamwork; local opinion leaders; existing networks; power and authority The category Innovation in i-PARIHS is similar to Evidence in PARIHS, but also incorporates innovation attributes (e.g. relative advantage and trialability)
Cabana et al., 1999 [37]	Physicians’ adherence to clinical practice guidelines	Based on analysis of 76 articles that identify barriers to adherence to “clinical practice guidelines, practice parameters, clinical policies, or national consensus statements” ([37], p. 1458)	10 categories (2 relate to contextual influences): • Lack of familiarity • Lack of awareness • Lack of agreement with specific guidelines • Lack of agreement with guidelines in general • Lack of outcome expectancy • Lack of self-efficacy • Lack of motivation/inertia of previous practice • Guideline factors • External barriers • Environmental factors Categories are referred to as “categories of barriers”	• External barriers: inability to reconcile patient preferences with guideline recommendations • Environmental factors: lack of time; lack of resources; organizational constraints (including insufficient staff or consultant support); lack of reimbursement; perceived increase in malpractice liability
Mäkelä and Thorsen, 1999 [43]	Implementation of guidelines to achieve practice change	Based on “previous work in the area” and data from various projects within a project called Concerted Action of the Changing Professional Practice ([44], p. 24)	3 categories (2 relate to contextual influences): • Professionals • Patients • Environment Categories are referred to as “barriers” and “facilitators”	• Patients: knowledge; skills; attitudes; other resources (including money and assistance) • Environment: Social factors (support for or discouragement of change by others, including colleagues, managers, opinion leaders, professional organizations and patients); organizational factors (including availability of guidelines at workplace and local infrastructures or rules and practicality within existing practice setting or routines); economic factors (including availability or lack of resources such as time and personnel)
Grol and Wensing, 2004 [39]	Achieving evidence-based practice	Based on “a summary of some of the theories and models relating to implementing change in diabetes care” ([39], p. S57)	3 categories (2 relate to contextual influences): • Individual professionals • Social context • Organizational and economic context Categories are referred to as “theories/models” and sub-categories as “factors”	• Social context: social learning (including incentives, feedback and reinforcement); social network and influence; patient influence; leadership • Organizational and economic context: innovativeness of organization (extent of specialization, decentralization, professionalization and functional differentiation); quality management (culture, leadership, organization of processes, customer focus); complexity (including interactions between parts of a complex system); organizational learning (capacity and arrangements for continuous learning in organization); economic (reimbursement arrangements, rewards, incentives)
Fleuren et al., 2004 [40]	Implementation of innovations in health care organizations	Based on analysis of 57 articles followed by a Delphi process involving 44 implementation experts	5 categories (4 relate to contextual influences): • Innovation • Socio-political context • Organization • Adopting person/user/health professional • Facilities needed to implement the innovation Categories are referred to as “determinants”	• Socio-political context: willingness of the patient to cooperate with the innovation; degree to which the patient is aware of the health benefits of the innovation; patient discomfort as a result of the innovation • Organization: organizational size; staff turnover; degree of staff capacity in the organization; nature of the collaboration between departments involved in the innovation • Adopting person/user/health professional: support from/of colleagues in implementing the innovation; support from/of other health professionals in implementing the innovation; support from/of their supervisors in the department/organization with respect to the implementation of the innovation; extent to which colleagues implement the innovation (modelling) • Facilities needed to implement the innovation: financial resources; reimbursement for health professionals/organizations; other resources; administrative support; time available; availability of staff; opinion leader
Greenhalgh et al., 2005 [23, 49]	Diffusion, dissemination and sustainability of innovations and delivery of health services	Based on analysis of 450 articles and books [23]; the model also consists of links between various determinants	7 categories (5 relate to contextual influences): • Innovations • Adopters and adoption • Diffusion and dissemination • Inner context • Outer context • Implementation and routinization • Linkage between components in the model Categories are referred to as “key topic areas”	• Diffusion and dissemination: network structure; homophily; opinion leaders; champions; boundary spanners (individuals with external ties); formal dissemination programmes • Inner context: structural determinants of innovativeness (e.g. the organization’s size, maturity, differentiation, specialization, slack resources and decentralization); absorptive capacity for new knowledge; receptive context for change (including leadership, strategic vision, managerial relations): tension for change; innovation-system fit (innovation fit with existing values, norms, strategies, goals, skill mix, etc.); assessment of implications; support and advocacy; dedicated time and resources; capacity to evaluate the innovation • Outer context: informal inter-organizational networks; intentional spread strategies; wider environment; political directives • Implementation and routinization: organizational structure; leadership and management; human resource issues; funding; intra-organizational communication; extra-organizational networks; feedback; adaptation/reinvention • Linkage between components in the model: linkage at development stage (of the innovation); role of change agency; external change agents Greenhalgh et al. [49] feature slightly different terms and categorizations
TDF: Michie et al., 2005 [48]; Cane et al., 2012 [13]	Behaviour change	Based on analysis of 33 behaviour change theories (encompassing 128 constructs)	14 categories of determinants (3 relate to contextual influences): • Knowledge • Skills • Beliefs about capabilities • Optimism • Beliefs about consequences • Reinforcement • Intentions • Goals • Memory, attention and decision process • Emotions • Behavioural regulation • Social/professional role and identity • Environmental context and resources • Social influences Categories are referred to as “domains” and sub-categories as “component constructs”	• Social/professional role and identity: professional identity; professional role; social identity; identity; professional boundaries; professional confidence; group identity; leadership; organizational commitment • Environmental context and resources: environmental stressors; resources/material resources; organizational culture/climate; salient events/critical incidents; person × environment interaction; barriers and facilitators • Social influences: social pressure; social norms; group conformity; social comparisons; group norms; social support; power; intergroup conflict; alienation; group identity; modelling
Wensing et al., 2005 [44]	Behaviour change	Based on analysis of the literature concerning theories on behaviour or organizational change in a variety of disciplines	4 categories (3 relate to contextual influences): • Individuals • Professional group • Health care organization • Economic structures Categories are referred to as “factors”	• Professional group: team cognitions; team processes; leadership and key individuals; social network characteristics; professional development • Health care organization: specification; flexibility; continuous improvement; external communication; internal communication; leadership structure; specialization; technical knowledge; organizational size • Economic structures: positive incentives; provider and patient financial risk sharing; transaction costs; purchaser-provider contract relationships; competition intensity; priority on social agenda.
AIF: Fixsen et al., 2005 [22]; Blase et al., 2012 [42]	Implementation of evidence-based interventions	Based on analysis of the diffusion and dissemination literature and the implementation literature in education and leadership	3 categories (2 relate to contextual influences): • Competency drivers • Organization drivers • Leadership drivers Categories are referred to as “implementation drivers”, which constitute “the infrastructure for implementation because they are the processes required to implement, sustain and improve identified effective interventions” ([43], p. 15–16). The category Competency drivers refers to training of staff, thus being more akin to implementation strategies	• Organization drivers: decision-support data systems; facilitative administration; systems intervention (including creating feedback loops concerning the implementation); the importance of organizational culture, climate and infrastructure is also mentioned in the description of this category • Leadership: no sub-categories are listed
NICS, 2006 [45]	Change in clinical practice	The basis for the identified determinant categories is not explicitly stated, but most likely existing literature	6 categories (4 relate to contextual influences): • The innovation itself • Individual professional • Patient • Social context • Organizational context • Economic and Political context Categories are referred to as “barriers”	• Patient: knowledge; skills; attitude; compliance • Social context: opinion of colleagues; culture of the network; collaboration; leadership • Organizational context: care processes; staff; capacities; resources; structures • Economic and political context: financial arrangements; regulations; policies
Cochrane et al., 2007 [14]	Optimal care, in terms of implementation of guidelines, evidence and research into practice	Based on analysis of 256 articles to respond to two research questions: how are barriers assessed and what types of barriers are identified?	7 categories (3 relate to contextual influences): • Cognitive/behavioural barriers • Attitudinal/rational-emotive barriers • Health care professional/physician barriers • Clinical practice guidelines/evidence barriers • Support/resource barriers • System/process barriers • Patient barriers Categories are referred to as “barriers” and sub-categories referred to as “categories”	• Support/resource barriers: time; support; human and material resources; financial resources • System/process barriers: organization and structure; teamwork structure and ethic; referral process • Patient barriers: patient characteristics; patient adherence The sub-categories are not fully explained in the framework or accompanying text (e.g. it is not obvious what is meant by “system” or “organizational” belonging to the “System/process barriers” category)
Nutley et al., 2007 [25]	Use of research	Based on analysis of “a wide range of studies” in the “factors affecting” literature ([25], p. 66–67)	4 categories (1 relate to contextual influences): • The nature of the research to be applied • The personal characteristics of both researchers and potential research users • The links between research and its users • The context for the use of research	No specific sub-categories are listed, but the following aspects are mentioned as important aspects of the context: lack of time; lack of professional autonomy to implement findings from research; local cultural resistance; lack of financial, administrative and personal support
PRISM: Feldstein and Glasgow (2008) [41]	Adoption, implementation and sustainability of health care interventions and programs	Based on analysis of “models in common use in implementation and diffusion research”, authors’ implementation experience, and concepts from the areas such as quality improvement, chronic care and Diffusion of Innovations	4 categories (all relate to contextual influences) • Program/intervention (organizational perspective and patient perspective) • Recipients (organizational characteristics and patient characteristics) • External environment • Implementation and sustainability infrastructure Categories are referred to as “domains”	Program/intervention: readiness; strength of the evidence base; coordination across departments and specialities; burden (complexity and cost); patient centeredness; patient choices; service and access; feedback of resultsRecipients: organizational health and culture; clinical leadership; management support and communication; systems and training; data and decision support; expectations of sustainability External environment: regulations; competition; reimbursement Implementation and sustainability infrastructure: dedicated team; bridge researchers; adaptable protocols and procedures; adopter training and support; plan for sustainability Both program/intervention and recipient categories consider staff at three levels: senior leaders, mid-level managers, and frontline workers
CFIR: Damschroder et al., 2009 [15]	Influences on implementation (outcomes)	Based on analysis of the 19 theories, frameworks and models used in implementation science	5 categories (3 relate to contextual influences): • Intervention characteristics • Characteristics of individuals • Process • Inner setting • Outer setting Categories are referred to as “domains” and sub-categories as “constructs”	• Process: planning; engaging (including opinion leaders and champions); executing; reflecting and evaluating (including feedback about the progress) • Inner setting: structural characteristics (including age maturity and size of the organization); networks and communications; culture; implementation climate (including absorptive capacity for change, tension for change, capability, relative priority, organizational incentives and rewards, learning climate); readiness for implementation (including leadership engagement, available resources, access to knowledge and information) • Outer setting: patient needs and resources; cosmopolitanism (networking with other external organizations); peer pressure (to implement an intervention); external policies and incentives
Gurses et al., 2010 [46]	Compliance with evidence-based guidelines	Based on analysis of 13 theories, models and frameworks used in implementation science (11 found through literature review and 2 identified by brainstorming)	4 categories (2 relate to contextual influences): • Clinician characteristics • Guideline characteristics • Implementation characteristics • System characteristics Categories are referred to as “categories” and sub-categories as “factors”	• Implementation characteristics: tension for change; mandate/preparation planning; leader and middle manager involvement and support; relative strength of supporters (including opinion leaders) and opponents; funding availability; monitoring and feedback mechanisms • System characteristics: task characteristics (including workload); tools and technologies (including available checklists as cognitive aids to facilitate work); physical environment (including layout, workspace and noise); organizational characteristics (including culture, teamwork, communication)
SURE: WHO, 2011 [47]	Implementation of policy options	Based on “published lists of barriers for implementing change in health care” ([48], p. 6), although it is not clear which these lists are	5 categories (4 relate to contextual influences): • Providers of care • Recipients of care • Other stakeholders • Health system constraints • Social and political constraints Categories are referred to as “barriers”	• Recipients of care: knowledge and skills; attitudes regarding programme acceptability, acceptability and credibility; motivation to change or adopt new behaviour • Other stakeholders: knowledge and skills; attitudes regarding programme acceptability, acceptability and credibility; motivation to change or adopt new behaviour • Health system constraints: accessibility of care; financial resources; human resources; educational system; clinical supervision; internal communication; external communication; allocation of authority; accountability; management or leadership; information systems; facilities; patient flow processes; procurement and distribution systems; incentives; bureaucracy; relationship with norms and standards • Social and political constraints: ideology; short-term thinking; contracts; legislation or regulations; donor policies; influential people; corruption; political stability
TICD: Flottorp et al., 2013 [34]	Improvements in health care professional practice	Based on analysis of 12 “checklists” described in implementation science (theories, frameworks and models)	7 categories of determinants (5 refer to contextual influences) • Guideline factors • Individual health professional factors • Patient factors • Professional interactions • Incentives and resources • Capacity for organizational change • Social, political and legal factors Categories are referred to as “domains of factors”	• Patient factors: patient needs; patient beliefs and knowledge; patient preferences; patient motivation; patient behaviour • Professional interaction: communication and influence; team processes; referral processes • Incentives and resources: availability of necessary resources; financial incentives and disincentives; nonfinancial incentives and disincentives; information system; quality assurance and patient safety systems; continuing education system; assistance for clinicians • Capacity for organizational change: mandate, authority, accountability; capable leadership; relative strength of supporters and opponents; regulations, rules, policies; priority of necessary change; monitoring and feedback; assistance for organizational changes • Social, political and legal factors: economic constraints on the health care budget; contracts; legislation; payer or funder policies; malpractice liability; influential people; corruption; political stability The category Guideline factors includes cultural appropriateness, i.e. congruity with customs and norms in the context of the implementation

The remaining 56 publications identified in the database searches were excluded because they reported applications of published determinant frameworks. However, the reference lists of those publications were examined to identify the original publications that described the development and contents of the frameworks. This inspection process yielded five determinant frameworks, which were included: Grol and Wensing [39]; Fleuren et al. [40]; Feldstein and Glasgow [41] (PRISM: Practical, Robust Implementation and Sustainability Model); Damschroder et al. [15] (CFIR: Consolidated Framework for Implementation Research); and Flottorp et al. [34] (TICD: Tailored Implementation for Chronic Diseases). Thus, the database searches resulted in the identification of eight unique determinant frameworks.

Inspection of the nine textbooks yielded three determinant frameworks that were not found in the database searches: Greenhalgh et al. [23]; Fixsen et al. [22], Blase et al. [42] (AIF: Active Implementation Frameworks); and Nutley et al. [25]. The five overviews identified six additional determinant frameworks not obtained by means of database searches or textbooks: Mäkelä and Thorsen [43]; Wensing et al. [44]; Rainbird et al. [45] (NICS: National Institute of Clinical Studies); Cochrane et al. [14]; Gurses et al. [46]; and WHO’s SURE (Supporting the Use of Research Evidence [47].

We included two publications each describing AIF [22, 42], TDF [13, 48] and the framework by Greenhalgh et al. [23, 49]: the first publication on the respective framework and a later publication that offered a more comprehensive description or refinement of the framework, thus warranting its inclusion. It should be noted that the TDF was not named so until Cane et al. [13]. Three publications concerning PARIHS were included; the first publication [11], a later publication [12], with a more comprehensive description of the framework, and a more recent publication featuring a revised version of the framework called integrated-PARIHS (i-PARIHS) [38], which was developed to “address a number of perceived limitations to its effective utilisation” ([38], p. 2). The framework by Grol and Wensing [39] is very similar to the one described by Grol et al. [24], but the former provides some more details, which is why we chose the first publication.

**Fig. 1:**
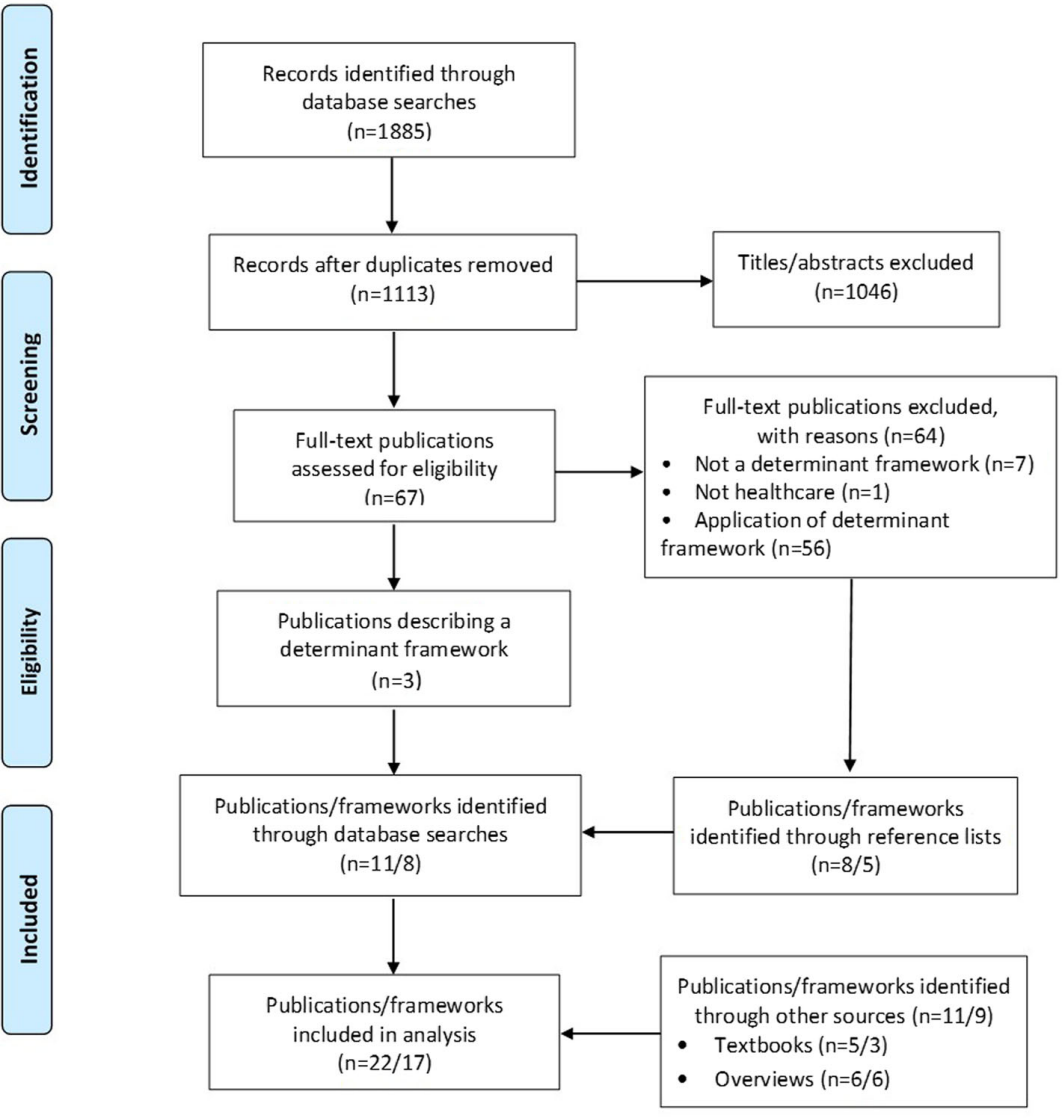
Identification and selection of publications and determinant frameworks

The selection process is illustrated in Fig. 1.

### How were the determinant frameworks developed?

The frameworks were developed in three different ways, as described in the investigated publications. Eleven frameworks are based on literature reviews of empirical studies and of theories, models and frameworks used in implementation science to describe determinants of various implementation-relevant outcomes: Cabana et al. [37]; Fleuren et al. [40]; Greenhalgh et al. [23]; Cochrane et al. [14]; Nutley et al. [25]; Feldstein and Glasgow (PRISM) [41]; Damschroder et al. (CFIR) [15]; Gurses et al. [46]; and Flottorp et al. (TICD) [34]. Presumably the framework by Rainbird et al. [45] is also based on a literature review, although details about how this framework was developed are not actually provided. The basis of the framework developed by SURE [47] is also somewhat unclear; it is simply stated that “published lists of barriers for implementing changes in health care often show a high degree of overlap” ([47], p. 6), implying that it was developed based on the existing literature. Fleuren et al. [40] combined the literature review with a Delphi process involving 44 implementation experts.

Four of the frameworks are based on the authors’ own implementation experiences and/or empirical studies. PARIHS [11] emerged from the observation that successful implementation in health care might be premised on three key determinants (characteristics of the evidence, context and facilitation), a proposition that was subsequently analysed in four empirical studies. PARIHS subsequently underwent substantial research and development work [12]. The revised i-PARIHS was proposed by Harvey and Kitson ([38], p. 2) based on their own “ongoing application of the framework in implementation studies together with critiques and evaluations of the framework by other research teams”. Grol and Wensing ([39], p. 558) based their work on “analyses of the literature and research conducted at our research centre”. Similarly, the AIF [22, 42] combined the developers’ implementation experiences with literature reviews. Mäkelä and Thorsen [43] referred to “previous work in the area” and data from various projects within a project called Concerted Action of the Changing Professional Practice ([43], p. 24).

Two frameworks are derived from existing theory or theoretical assumptions rather than experience or empirical studies. The framework by Wensing et al. [44] was based on an analysis of the literature concerning theories on behaviour or organizational change in a variety of disciplines. It is not stated how many theories were identified, but the searches continued “until the overview of theories was ‘saturated’” ([44], p. 16). The TDF [13] was constructed on the basis of a synthesis of 128 constructs related to behaviour change found in 33 established social-cognitive behaviour change theories.

### What terms are used to denote contextual determinants?

Six of the 17 frameworks explicitly refer to “context” as a contextual determinant category [11, 12, 23, 25, 38–40, 45]. The other 11 frameworks use a broad range of terms to denote various contextual determinants, including terms such as “external barriers” [37], “environmental factors” [37], “environment” [43], “external environment” [41], “inner setting” and “outer setting” [15], “system characteristics” [46] and “organizational drivers” [42].

### How is context conceptualized?

Most of the 17 frameworks do not provide specific definitions of context. Instead, they define the concept indirectly by describing a number of contextual determinants (categories and/or sub-categories) that together make up the context. Three frameworks [11, 13, 15] provided a specific definition of the context concept.

The CFIR [15] is presented in a paper that provides a definition of context although the framework itself refers to “inner and outer setting” rather than context: “Context consists of a constellation of active intervening variables and is not just a backdrop for implementation. … For implementation research, ‘context’ is the set of circumstances or unique factors that surround a particular implementation effort … In this paper, we use the term context to connote this broad scope of circumstances and characteristics” ([15], p. 3).

The TDF includes one category, “environmental context and resources”, that explicitly refers to context. This category is defined as “any circumstance of a person’s situation or environment that discourages or encourages the development of skills and abilities, independence, social competence and adaptive behaviour” ([13], p. 14).

Kitson et al. ([11], p. 150] define context in relation to PARIHS as “the environment or setting in which the proposed change is to be implemented”. The revised version of PARIHS, i-PARIHS, has a wider focus on the different layers of context, differentiating between the immediate local level, the wider organizational level and external health system level, something that was not done in the original PARIHS [38].

### What context dimensions are included in the frameworks?

Contextual determinants in the 17 frameworks were categorized into 12 different context dimensions (Table 2). The most comprehensive framework was PRISM [41], which included contextual determinants that could be mapped to 11 context dimensions (Table 3). It was followed by PARIHS [11, 12, 38], CFIR [15], TICD [34] and the framework by Greenhalgh et al. [23], all of which included contextual determinants that could be mapped to ten context dimensions.

**Table 2:** Description of the context dimensions

Context dimension	Description
Micro level of health care	
Patients	Patients’ preferences, expectancies, attitudes, knowledge, needs and resources that can influence implementation
Meso level of health care	
Organizational culture and climate	Shared visions, norms, values, assumptions and expectations in an organization that can influence implementation (i.e. organizational culture) and surface perceptions and attitudes concerning the observable, surface-level aspects of culture (i.e. climate).
Organizational readiness to change	Influences on implementation related to an organization’s tension, commitment or preparation to implement change, the presence of a receptive or absorptive context for change, the organization’s prioritization of implementing change, the organization’s efficacy or ability to implement change, practicality and the organization’s flexibility and innovativeness
Organizational support	Various forms of support that can influence implementation, including administration, planning and organization of work, availability of staff, staff workload, staff training, material resources, information and decision-support systems, consultant support and structures for learning
Organizational structures	Influences on implementation related to structural characteristics of the organization in which implementation occurs, including size, complexity, specialization, differentiation and decentralization of the organization
Macro level of health care	
Wider environment	Exogeneous influences on implementation in health care organizations, including policies, guidelines, research findings, evidence, regulation, legislation, mandates, directives, recommendations, political stability, public reporting, benchmarking and organizational networks
Multiple levels of health care	
Social relations and support	Influences on implementation related to interpersonal processes, including communication, collaboration and learning in groups, teams and networks, visions, conformity, identity and norms in groups, opinion of colleagues, homophily and alienation
Financial resources	Funding, reimbursement, incentives, rewards, costs and other economic factors that can influence implementation
Leadership	Influences on implementation related to formal and informal leaders, including managers, key individuals, change agents, opinion leaders, champions, etc.
Time availability	Time restrictions that can influence implementation
Feedback	Evaluation, assessment and various forms of mechanisms that can monitor and feed back results concerning the implementation, which can influence implementation
Physical environment	Features of the physical environment that can influence implementation, e.g. equipment, facilities and supies

**Table 3:** Context dimensions addressed in the frameworks

Context dimensions	PARIHS [11, 12, 38]	Cabana et al. [37]	Mäkelä and Thorsen [43]	Grol and Wensing [39]	Fleuren et al. [40]	Greenhalgh et al. [23]	TDF [13, 48]	Wensing et al. [44]	AIF [22, 42]	NICS [45]	Cochrane et al. [14]	Nutley et al. [25]	PRISM [41]	CFIR [15]	Gurses et al. [46]	SURE [47]	TICD [34]	Number of frameworks that address the context dimension
Organizational support	✓	✓	✓	✓	✓	✓	✓	✓	✓	✓	✓	✓	✓	✓	✓	✓	✓	17
Financial resources	✓	✓	✓	✓	✓	✓	✓	✓		✓	✓	✓	✓	✓	✓	✓	✓	16
Social relations and support	✓		✓	✓	✓	✓	✓	✓		✓	✓	✓	✓	✓	✓	✓	✓	15
Leadership	✓		✓	✓	✓	✓	✓	✓	✓	✓			✓	✓	✓	✓	✓	14
Organizational culture and climate	✓			✓		✓	✓	✓	✓			✓	✓	✓	✓	✓	✓	12
Organizational readiness to change	✓		✓	✓		✓	✓	✓	✓			✓	✓	✓	✓		✓	12
Organizational structures	✓		✓	✓	✓	✓		✓	✓	✓	✓		✓	✓				11
Patients	✓	✓	✓	✓	✓					✓	✓		✓	✓		✓	✓	11
Wider environment	✓				✓	✓	✓	✓		✓			✓	✓		✓	✓	10
Feedback	✓			✓		✓			✓				✓	✓	✓		✓	8
Time availability		✓	✓		✓	✓					✓	✓	✓					7
Physical environment															✓		✓	2
Number of context dimensions	10	4	8	9	8	10	7	8	6	7	6	6	11	10	8	7	10	

In Nutley et al. [25], “Lack of professional authority to implement findings from research” was categorized as organizational readiness to change. Organizational culture in Greenhalgh et al. [23] is not explicitly listed as a sub-category of “inner context” (although organizational climate is), but the authors state that the inner context comprises both “the ‘hard’ medium of visible organizational structure and the ‘soft’ medium of culture” ([23], p. 134)

The 12 context dimensions pertain to different levels of aggregation, from the micro to the macro level of health care. At the micro level, patients can influence implementation. Four broader organizational determinants can be attributed to the meso level: organizational culture and climate, organizational readiness to change, organizational structures, and organizational support. The macro level consists of even broader, “outside”, influences from the wider environment. It was not possible to attribute six of the context dimensions to a single level of aggregation because they may affect both the micro and meso levels (and to some extent also the macro level): social relations and support, financial resources, leadership, time availability, feedback and physical environment.

The most common context dimensions were organizational support (included in all 17 frameworks), financial resources (16 frameworks), social relations and support (15 frameworks), leadership (14 frameworks), organizational culture and climate (12 frameworks) and organizational readiness to change (12 frameworks). The least common dimension was physical environment (2 frameworks). Patients as a contextual determinant was addressed in 11 of the frameworks.

## Discussion

This scoping review identified 17 unique frameworks in implementation science that address contextual determinants. The results show there is considerable variation with regard to the terms used to denote contextual determinants, how context is defined and conceptualized, and which contextual determinants are accounted for. Most of the frameworks were developed based on empirical studies and theories, models and frameworks used in implementation science to describe determinants of implementation outcomes. Hence, there is considerable intra-field referencing, as many researchers have developed frameworks partially based on earlier frameworks. This could potentially contribute to consolidation and convergence towards a number of core context dimensions, but it could also lead to a less inductive approach to exploring and understanding the context.

Interestingly, most of the frameworks do not actually mention or refer to “context”, instead using other terms to denote such determinants. Furthermore, few of the frameworks provide a precise definition or clarify the meaning of the concept. Instead, most frameworks define the concept indirectly, in terms of specifying a number of determinants that comprise the context. These differences notwithstanding, it is clear that context is commonly viewed as a multi-dimensional concept. The frameworks differed with regard to how many and which determinant categories were related to context (from one contextual determinant category to five) and the proportion of context categories in relation to all determinant categories. In most frameworks, context is one of several determinants and a relatively minor aspect. For instance in the TDF [13], only three of the 14 determinant categories relate to contextual determinants. In some frameworks context is a more important aspect; in PRISM [41], all four determinant categories relate to contextual determinants, and in the framework by Fleuren et al. [40], four of five determinant categories account for contextual determinants.

We found a large variation in the number of contextual determinants (i.e. categories and sub-categories) described in the frameworks. For example, Gurses et al. [46] list 10 sub-categories belonging to two categories of contextual determinants, whereas Greenhalgh et al. [23] provide a list of 22 sub-categories that are part of five contextual determinant categories. Frameworks such as those by Greenhalgh et al. [23], CFIR [15] and TICD [34] are quite specific and detailed concerning the contextual determinants. Some of the differences with regard to the number of contextual determinants are due to slightly different aims of the frameworks. Although all frameworks address influences on implementation, the focus varies somewhat, with some identifying determinants for behaviour change and others describing determinants pertaining to adherence to guidelines, research use or use of innovations.

The frameworks broadly include two types of context dimensions: those that function as necessary conditions for implementation and those that may be viewed as active, driving forces required to achieve successful implementation. For instance, having sufficient financial resources and time availability may constitute favourable conditions for implementation, but they likely need to be combined with, for example, supportive leadership and social relations if implementation is to succeed. This means that strategies to facilitate implementation, which are usually described as a determinant category of its own [10], overlap with some context dimensions. Implementation strategies have been defined as “methods or techniques used to enhance the adoption, implementation and sustainability of a clinical program or practice” [50]. Hence, the boundary between implementation strategies and some contextual determinants on implementation is ambiguous. One of the dimensions, readiness for change, differs from the others since it is specific to the (evidence-based) practice being implemented, whereas the other context dimensions have relevance regardless of specific practices.

The frameworks describe discrete contextual determinants by breaking down context into a number of constituent parts. However, the 12 context dimensions are interdependent. For instance, a lack of available staff (organizational support) and/or poor funding for the implementation (financial resources) will likely have a negative impact on the organization’s preparedness for implementation (organizational readiness to change). Therefore, it is important to view context in holistic terms because successful implementation depends on combinations of different contextual determinants. Taking an overly reductionist approach, studying the impact of different dimensions in isolation of each other neglects the fact that two or more seemingly unimportant contextual determinants may combine to create powerful effects, or potentially strong determinants may combine to generate weak effects. Stressing a holistic view, organizational behaviour theorist Johns [51] has referred to context as a “bundle of stimuli” and talked about “deadly combinations” of otherwise effective determinants that can yield unfavourable outcomes.

With regard to the most common context dimensions that emerged from the content analysis of the frameworks, most of the frameworks described contextual determinants that could be attributed to organizational support, financial resources, social relations and support, leadership and organizational culture and climate. Many of the barriers for implementation of evidence-based practices that have been identified earlier in the literature have been associated with these context dimensions [25, 52–55], underscoring their importance for understanding and addressing implementation challenges.

All the frameworks included some form of organizational support as a contextual determinant. This support was reflected in various types of administrative, technological and human resources that provide favourable conditions for successful implementation, e.g. planning and organization of work, availability of staff, staff training and information and decision-support systems. Organizational support has been associated with both attitudes toward EBP and EBP use in practice, and has also been shown to mediate the link between organization type (private vs. public organization) and attitudes toward EBP [56].

The dimension of financial resources, which was identified in all but one determinant framework, was expressed in terms of funding, reimbursement, incentives, rewards and costs, i.e. available economic means to support implementation. The importance of this context dimension is supported by a recent systematic review that found lack of financial resources to be an important barrier for the implementation of mental health services integration into primary health care [57]. Another study highlighted the importance of financial resources when implementing sustainability initiatives in health care facilities, particularily in those that are small and medium-sized [58]. These are just a few examples; this context dimension is obviously paramount when it comes to enabling almost any kind of implementation of change in a health care practice.

 Social relations and support was also a common context dimension, being comprised of various interpersonal processes that occur when the actions of one or more individuals influence the behaviour, attitudes or beliefs of one or more other individuals. This influence was described in the determinant frameworks as communication, collaboration and learning in groups, teams and networks, identity and norms in groups, and opinion of colleagues.

 Although most frameworks specifically refer to organizational culture, it is important to recognize that health care organizations are inherently multi-cultural given the variety of professions, departments, and teams operating within them [59, 60]. Indeed, it has increasingly been acknowledged that organizations rarely possess a single, homogeneous culture, and many organization theorists have questioned the overemphasis on “organizational” culture [61]. Professional cultures are particularly important in health care because professional groups differ with regard to values, norms, beliefs and behaviours [62]. It has been shown that professional groups can serve as barriers to implementation of evidence-based practices. For instance, Ferlie et al. [63] and Fitzgerald and Dopson [64] identified boundaries between different professional groups that inhibited the spread of new practices. Other studies have shown that professional loyalties may be stronger than those to the organization, which may impede change initiatives and implementation endeavours [65–69].

The emphasis on the organization rather than professions is likely due to implementation researchers being more influenced by organization research than the sociology of professions. Although none of the frameworks refer specifically to professional culture, several address social relations and group influences that may serve a similar function in potentially “over-ruling” individuals’ attitudes, beliefs and other behavioural predictors, e.g. “group norms” [13], “group identity” [13] and “culture of the network” [45]. While addressing organizational culture, two of the frameworks, AIF [22] and CFIR [15], also refer to various aspects of organizational climate, which is understood as the surface perceptions and attitudes concerning the observable, surface-level aspects of culture at a particular point in time [70]. Organizational climate is often defined as the employees’ perceptions of the impact of their work environment, taking into account aspects such as what is promoted, rewarded or punished in the work setting [71].

 Most of the frameworks refer to contextual determinants in terms of leadership rather than of management. A review of 17 studies that concerned the importance of leadership for implementation found that the two concepts tend to be used interchangeably and are rarely differentiated in implementation research [72]. However, whereas leadership is concerned with setting a direction for change and developing a vision for the future, management consists of realizing those goals through planning, budgeting and coordinating [73, 74]. Leadership is broader than management because it involves influence processes with a wide range of people, not just those who have a managerial role [75]. Hence, a research challenge to account for the importance of leadership is to identify and gather information from and about those who are leaders. Informal leaders often have a critical role in health care, e.g. clinicians whose views are highly regarded and who are particularly persuasive with their colleagues. Informal leaders may lead others in resisting implementation or change proposed by others [76–78].

Eleven of the 17 frameworks included patient-related determinants. The relatively low proportion is somewhat surprising in view of the growing importance of patient participation in health care policy making, practice and research [79]. Patient participation (and related concepts such as shared decision making, patient engagement and patient involvement) has been associated with improved health outcomes and has been advocated as a means to improve the quality of care [80, 81]. However, implementation science thus far has not emphasized research concerning potential patient determinants on implementation outcomes.

The 12 context dimensions that emerged from the content analysis of the determinant frameworks belong to different levels of aggregation, suggesting a multi-layered ecological model of the context. Ecological models are used in many disciplines and fields, e.g. public health, sociology, biology, education and psychology, to describe determinants at multiple levels, from the individual to society [82, 83]. Several of the context dimensions that we identified are multi-level and may influence implementation at different levels. This conceptualization of the context underscores that strategies to facilitate implementation must address more than one level. In line with this ecological model of context, some of the frameworks distinguish between an inner and an outer context of implementation. The inner context is typically understood as micro- and meso-level influences, whereas the outer context refers to macro-level influences beyond the organization, e.g. national guidelines, policies or collaboration with other organizations. Still, the “line” between inner and outer context is somewhat arbitrary and not always clear [15].

The fact that relatively few frameworks address the outer context (wider environment) indicates an emphasis on determinants that exist at organizational and lower-aggregate levels (e.g. teams or groups). Whereas “thick descriptions” of the wider circumstances of the implementation are valuable for interpreting findings, it may be difficult to capture or establish causality between the outer context and implementation outcomes. May et al. [9] argue that such a “whole system” approach makes it almost impossible to disentangle the complicated relationships between various determinants and to identify the causal mechanisms by which different processes and actors at multiple levels influence each other. This scepticism is relevant and points to the importance of identifying and accounting for key context dimensions in individual studies. Nevertheless, implementation scientists have focused primarily on the individual and organizational levels. While implementation science is a young field, its future development would benefit from drawing from other disciplines which have dealt more with the impact of the macro system, e.g. political science, prevention science, and complexity science.

The literature on implementation context has suggested that there are two different context conceptualizations: context as something concrete and passive, e.g. the physical environment in which implementation occurs; and context as something abstract but potentially dynamic, e.g. active support from colleagues and management [15, 46]. Most of the frameworks identified in this review emphasize the active view of context, indicating that it is widely recognized that context is not merely a passive backdrop to implementation. The view of context as a physical place implies a positivist notion of context, i.e. the context is an objective entity that can be observed, whereas the view of the context as something more intangible and active represents a more subjectivist perspective that acknowledges the complexity and multi-dimensionality of the context.

Organization theorists [84, 85] have described context as a socially constructed phenomenon that is difficult to manipulate or manage. However, the underlying assumption of the frameworks instead is that it is possible to break down the context into its constituent parts, which can be influenced to have an impact on implementation outcomes on the premise of a cause-and-effect relationship between the context and outcomes. Furthermore, some of the frameworks have spawned instruments to measure and quantify various aspects of the context, underscoring an essentially objectivist understanding of the context in implementation science. Examples of such instruments are the Alberta Context Tool [86] and the Context Assessment Index [87].

A few recently published reviews have also attempted to identify determinant frameworks, but have used different, albeit overlapping, selection criteria and research questions. Li et al. [88] focused on organizational contextual determinants for the implementation of evidence-based practices in health care and identified six such determinants. All six of those determinants were included among the 12 context dimensions we identified in our review. While the review by Li et al. was limited to the organizational (meso) level, our review also identified contextual determinants at both micro and macro levels, including patients and the wider environment. A review by Strifler et al. [89] identified 159 different theories, models and frameworks, but they did not distinguish between the different types of theoretical approaches and did not delve into whether or how context was addressed. Their focus was on the use of the theories, models and frameworks in practice and research concerning prevention and/or management of cancer or other chronic diseases.

Discussion about the meaning and relevance of context is not unique to implementation science. Researchers in quality improvement have defined context as “everything else that is not the intervention” ([90], p. 605). This is somewhat similar to implementation science, in that the intervention, e.g. an evidence-based practice, is not considered to be part of the context. However, researchers in implementation science typically view this “everything else” in terms of characteristics of the adopters (e.g. health care professionals) and the strategies used to support the implementation. In organizational behaviour, context is typically understood as influences that are external to and/or “above” (i.e. a higher aggregation level than) the individual, e.g. a team, professional group, department or organization [91, 92]. This perspective of context resembles the view conveyed in the implementation science frameworks in this review.

In the learning literature, context is considered to be “multisensory, diffuse and continuously present” ([93], p. 418). Various forms of context have been described, including spatial context (everything we do occurs in a place), temporal context (events are often defined by their sequential properties), cognitive context (influences how information is perceived, processed and stored), and social and cultural contexts (influence how we understand the world and ourselves) [94–97]. The temporal aspect of context was not explicitly addressed in any of the frameworks in this review other than time being considered a limited resource (time availability). However, it seems obvious that the timing of implementation could have an impact on the outcomes. For instance, successful results seem less likely if the implementation of an evidence-based practice coincides with numerous other change initiatives or if it occurs during a time of change fatigue, i.e. feelings of stress, exhaustion and burnout among staff associated with rapid and continuous changes in the workplace [98]. Although not explicitly mentioned in any of the frameworks, the timing of implementation may be considered an underlying influence on time availability and organizational readiness to change.

### Study limitations

Some limitations of this review should be acknowledged. We only searched two databases and we may not have identified all relevant determinant frameworks. Although our searches yielded thousands of hits, most publications were excluded because they did not describe a determinant framework according to our definition. Our focus on health care settings may have led us to miss relevant frameworks used in other fields, such as public health, community-based services, and in disciplines such as psychology, sociology, organizational theory and political science, which limits the generalizability of our findings. We did not attempt any kind of quality assessment of the included publications or frameworks. This was not considered feasible due to the variety in study design and scope of the different publications.

## Conclusions

This scoping review of 17 determinant frameworks in implementation science shows that there is considerable variation with regard to how the frameworks were developed, the terms used to denote contextual determinants, how context is defined and conceptualized, and which contextual determinants are accounted for in frameworks used in implementation science. Most of the included frameworks provide only a limited and narrow description and definition of context, and a broad range of terms is used to denote various contextual determinants. Context is generally not described consistently, coherently or comprehensively in determinant frameworks, and there is inconsistency with regard to which contextual determinants are addressed. Still, it was possible to identify common dimensions of the context based on the frameworks, the most frequently used being organizational support, financial resources, social relations and support, leadership, and organizational culture and climate.

Our categorization of context dimensions may help the implementation researcher to consider the relevance of the various determinants in a structured way. Ultimately, however, the findings of this review are consistent with the observation by Pfadenhauer et al. ([8], p. 104) that context in implementation science is an “inconsistently defined and applied” concept that is “only partially mature”.

It is important that researchers are aware of how context is defined or interpreted in studies, which context dimensions are considered, and why these dimensions might be relevant. The challenge for the researcher is to identify the most important context dimensions and address these in the research. Although it is difficult to capture all potentially relevant influences in any given study, recognition of core context dimensions can facilitate research that incorporates a theory of context, i.e. assumptions about how different dimensions may influence each other and affect implementation outcomes. A thoughtful application of the concept and a more consistent terminology will enhance transparency, simplify communication among researchers, and facilitate comparisons across studies. Together, these advances will further our understanding of the role of context within implementation science.

## Additional file

Additional file 1: Search strategy and results. Presentation of search strategies and number of records identified in the database searches. (DOCX 18 kb)

## Abbreviations

AIF: Active Implementation Frameworks
CFIR: Consolidated Framework for Implementation Research
NICS: National Institute of Clinical Studies
PARIHS: Promoting Action on Research Implementation in Health Services
PRISM: Practical, Robust Implementation and Sustainability Model
SURE: Supporting the Use of Research Evidence
TDF: Theoretical Domains Framework
TICD: Tailored Implementation for Chronic Diseases
