# Supplementary material for: Context matters in implementation science: a scoping review of determinant frameworks that describe contextual determinants for implementation outcomes
Source: BMC Health Serv Res. 2019 Mar 25;19:189. doi: 10.1186/s12913-019-4015-3 (PMC6432749; doi:10.1186/s12913-019-4015-3)
Supplement: Supplementary file 1 — Search strategy and results. Presentation of search strategies and number of records identified in the database searches. (DOCX 18 kb) [file 12913_2019_4015_MOESM1_ESM.docx]

**Additional file 1** Search strategies and results

1. **Ovid/MEDLINE, 9 Nov 2017**

| 1 | (determinant* or barrier* or hinder* or obstacle* or impediment* or enabl* or facilitat* or moderat* or mediat* or modif* or drive* or factor* or influenc* or impact* or domain* or construct*).ab,ti. | 7916771 |
| --- | --- | --- |
| 2 | exp Communication Barriers/ | 6254 |
| 3 | 1 or 2 | 7919725 |
| 4 | (theor* or framework* or model* or taxonom* or classifi* or categor* or organiz* or organis* or checklist* or concept*).ab,ti. | 4446387 |
| 5 | exp Classification/ | 201746 |
| 6 | exp Checklist/ | 5272 |
| 7 | 4 or 5 or 6 | 4581284 |
| 8 | 3 adj3 7 | 2006138 |
| 9 | (context* or setting* or environment* or system*).ab,ti. | 1851623 |
| 10 | (implementation or implementing or knowledge translation or knowledge transfer or knowledge-to-action or improvement or improving).ab,ti. | 779168 |
| 11 | exp Health Plan Implementation/ | 5339 |
| 12 | 10 or 11 | 781115 |
| 13 | (guideline* or adherence or compliance or adoption or uptake or "research use" or "research utilization" or "routine embedding" or practice or performance or "behaviour change" or "behavior change”).ab,ti. | 2162142 |
| 14 | exp Guideline Adherence/ | 30060 |
| 15 | 13 or 14 | 2170903 |
| 16 | (health or health care or medicine or clinical).ab,ti. | 1962699 |
| 17 | 8 and 9 and 12 and 15 and 16 | 972 |
| 18 | limit 17 to English language | 957 |

**Ovid/Embase, 9 Nov 2017**

| 1 | (determinant* or barrier* or hinder* or obstacle* or impediment* or enabl* or facilitat* or moderat* or mediat* or modif* or drive* or factor* or influenc* or impact* or domain* or construct*).ab,ti. | 9094009 |
| --- | --- | --- |
| 2 | (theor* or framework* or model* or taxonom* or classifi* or categor* or organiz* or organis* or checklist* or concept*).ab,ti. | 5024489 |
| 3 | exp Classification/ | 1647403 |
| 4 | exp Checklist/ | 16788 |
| 5 | 2 or 3 or 4 | 4748388 |
| 6 | 1 adj3 5 | 2270387 |
| 7 | (context* or setting* or environment* or system*).ab. | 2000473 |
| 8 | (implementation or implementing or knowledge translation or knowledge transfer or knowledge-to-action or improvement or improving).ab. | 937915 |
| 9 | (guideline* or adherence or compliance or adoption or uptake or "research use" or "research utilization" or "routine embedding" or practice or performance or "behaviour change" or "behavior change”).ab. | 2319239 |
| 10 | (health or health care or medicine or clinical).ab. | 2030972 |
| 11 | 6 and 7 and 8 and 9 and 10 | 941 |
| 12 | limit 11 to English language | 928 |
